# A Novel Insertion in the Hepatitis B Virus Surface Protein Leading to Hyperglycosylation Causes Diagnostic and Immune Escape

**DOI:** 10.3390/v15040838

**Published:** 2023-03-25

**Authors:** Felix Lehmann, Heiko Slanina, Martin Roderfeld, Elke Roeb, Jonel Trebicka, John Ziebuhr, Wolfram H. Gerlich, Christian G. Schüttler, Bernhard Schlevogt, Dieter Glebe

**Affiliations:** 1 Institute of Medical Virology, National Reference Center for Hepatitis B Viruses and Hepatitis D Viruses, German Center for Infection Research (DZIF; Partner Site Giessen-Marburg-Langen), Justus Liebig University, 35392 Giessen, Germany; felix.lehmann@viro.med.uni-giessen.de (F.L.); heiko.slanina@viro.med.uni-giessen.de (H.S.); john.ziebuhr@viro.med.uni-giessen.de (J.Z.); wolfram.h.gerlich@viro.med.uni-giessen.de (W.H.G.); christian.schuettler@viro.med.uni-giessen.de (C.G.S.); 2 Department of Gastroenterology, Justus Liebig University, 35392 Giessen, Germany; martin.roderfeld@innere.med.uni-giessen.de (M.R.); elke.roeb@innere.med.uni-giessen.de (E.R.); 3 Department of Medicine B, University Hospital Muenster, 48149 Muenster, Germany; jonel.trebicka@ukmuenster.de (J.T.); bernhard.schlevogt@klinikum-os.de (B.S.); 4 Department of Gastroenterology, Medical Center Osnabrueck, 49076 Osnabrueck, Germany

**Keywords:** hepatitis B virus, N-linked glycosylation, diagnostic escape, immune escape

## Abstract

Chronic hepatitis B virus (HBV) infection is a global health threat. Mutations in the surface antigen of HBV (HBsAg) may alter its antigenicity, infectivity, and transmissibility. A patient positive for HBV DNA and detectable but low-level HBsAg in parallel with anti-HBs suggested the presence of immune and/or diagnostic escape variants. To support this hypothesis, serum-derived HBs gene sequences were amplified and cloned for sequencing, which revealed infection with exclusively non-wildtype HBV subgenotype (sgt) D3. Three distinct mutations in the antigenic loop of HBsAg that caused additional N-glycosylation were found in the variant sequences, including a previously undescribed six-nucleotide insertion. Cellular and secreted HBsAg was analyzed for N-glycosylation in Western blot after expression in human hepatoma cells. Secreted HBsAg was also subjected to four widely used, state-of-the-art diagnostic assays, which all failed to detect the hyperglycosylated insertion variant. Additionally, the recognition of mutant HBsAg by vaccine- and natural infection-induced anti-HBs antibodies was severely impaired. Taken together, these data suggest that the novel six-nucleotide insertion as well as two other previously described mutations causing hyperglycosylation in combination with immune escape mutations have a critical impact on in vitro diagnostics and likely increase the risk of breakthrough infection by evasion of vaccine-induced immunity.

## 1. Introduction

With an estimated 296 million people chronically infected with Hepatitis B virus (HBV), HBV infection remains one of the leading causes of liver diseases including cirrhosis and hepatocellular carcinoma (HCC) [1]. HBV is most commonly transmitted during childbirth by an infected mother (vertical transmission), sexual contact (horizontal transmission), and contaminated medical equipment [2]. HBV is a partially double-stranded DNA virus that is enveloped by a host-derived membrane. Attachment and entry of HBV to host hepatocytes are enabled by three C-terminally redundant viral surface proteins embedded in this envelope: the small, middle, and large HBV surface proteins (SHBs, MHBs, and LHBs, respectively) collectively termed HBsAg. The 226 amino acid (aa)-long SHBs consists of the S domain with four putative transmembrane domains (TMD I-IV). The S domain harbors the major hydrophilic region (MHR; aa100-163) located between TMD II and III [3], which is essential for viral attachment to heparan sulfate-proteoglycans [4,5] on the hepatocyte membrane and harbors major neutralizing B-cell epitopes [6,7]. The ***a*** determinant (aa124-147), which is common to all HBsAg subtypes, is located within the MHR [8] and is the prime target for neutralizing antibodies during natural infection and following vaccination. SHBs has an obligate glycosylation site at N146 and is found in equal parts in its unglycosylated (p24) and glycosylated form (gp27) in serum [9]. Both forms have distinct functions in the viral life cycle: While p24 is important for cellular attachment, gp27 mainly acts as a glycan shield to mask immunogenic epitopes while also improving particle secretion [9,10]. MHBs contains an additional N-terminal domain (pre-S2; 55aa) [11] which may act as a spacer between the S domain and the pre-S1 but is not essential for the infectivity of HBV [12]. Its expression in patients is associated with highly replicative HBV infections [13,14]. LHBs is a major component of the HBV envelope and contains at its N-terminus the pre-S1 domain (108, 118, or 119aa depending on the HBV genotype) [15]. The pre-S1 domain is essential for HBV envelopment [16] and for infectivity [17] due to attachment to the HBV entry receptor NTCP [18]. While also essential for envelopment of virions [16], most of the SHBs protein forms non-infectious subviral particles in vast excess [15].

Since 1982, a recombinant vaccine consisting of yeast-expressed SHBs has been available and leads to excellent seroprotection rates of up to 99% in certain cohorts, e.g., in young women [19]. Even though the vaccine has proven to be highly effective, breakthrough infections and reactivation of immune escape variants are regularly described [20,21,22]. Vaccine- and passive immunoprophylaxis-associated escape mutations are often located between aa137 and 147, with G145R being most frequently reported [8,23]. While single amino acid substitutions in the S protein (such as K141E and G145R) can be sufficient to escape neutralizing antibodies against SHBs (anti-HBs), they often concomitantly cause a decrease in virion secretion efficiency [10,24,25]. Furthermore, substitutions and insertions that lead to novel N-linked glycosylation motifs (sequons; N-X-S/T) have been frequently reported [26,27]. The functionality of these sequons as N-glycosylation targets depends on their positions in the protein, neighboring residues, and secondary/tertiary structure. For the MHR within SHBs, sequon insertions/substitutions are most frequently observed between aa112 and 117 and between aa129 and 131 [27,28,29]. Glycosylation mutants may emerge individually or in combination with immune escape mutations [9,24,26,27,30,31,32] where they have been shown to alleviate the secretion deficit phenotype [10,24,26,27]. Of clinical concern, the N-glycosylation variants Q129N and T131N [26] as well as immune escape mutations such as D144E and G145R [33,34,35] have been shown to be transmissible horizontally.

Current diagnostic assays to detect active HBV infections and to monitor their progression rely mainly on the assay of HBsAg and, after seroconversion, anti-HBs in combination with measurements of HBV DNA levels. HBV variants that are associated with immune escape can similarly affect antibody-based diagnostic detection of HBsAg by masking or obscuring the epitopes targeted by the assay leading to a (false) “occult HBV infection (OBI)” phenotype (HBsAg negativity, despite detectable circulating HBV DNA) [36]. Here, we describe and characterize co-circulating HBV variants from a patient who was initially considered to have OBI but had very low levels of HBsAg and coexisting seemingly protective levels of anti-HBs. We found a complex pattern of mutations within the HBs antigenic loop, leading to hyperglycosylation of HBsAg and subsequent diagnostic and vaccine escape.

## 2. Materials and Methods

### 2.1. Patient Data

A patient was referred to the liver outpatient clinic of Muenster University Hospital due to a suspicion of occult HBV infection. Clinical data were retrieved from electronic patient records. HBV DNA levels and qualitative and quantitative HBsAg, anti-HBs, and anti-HBc in serum were determined using the Siemens Atellica MDX 160, the Abbott ARCHITECT, and the Roche Cobas e801 systems.

The patient samples were obtained in accordance with the ethics committee of Muenster University Hospital (AZ 2010-192-f-S). The patient was informed regarding the use of data and written informed consent was obtained.

### 2.2. Amplification and Cloning of HBV Sequences

The region encompassing the C-terminal region of SHBs covering codons 80 to 226 including the MHR was amplified via nested PCR by combining two primer sets previously published [37,38]. The amplification product was cloned into pcDNA3.1(+) via FastCloning as described [39]. In short, the pcDNA3.1(+) backbone was amplified using the primers 5′-CTA TTG ATT GGA AAG TTT GTC AAA Gct gca gat atc cag cac agt g-3′ and 5′-GCC GCA GAC ACA TCC Act gga cta gtg gat ccg agc tc-3′ to introduce homologous overhangs (capital letters) to the HBV amplification product. pcDNA3.1(+) backbone and HBV DNA insert were recombined and transformed into *E. coli* Stellar (Takara). Individual bacterial clones were picked to inoculate overnight cultures. Plasmid DNA was extracted using the Monarch Plasmid Miniprep kit (NEB) and Sanger sequenced (LGC genomics).

For the expression of variant SHBs, partial HBV sequences were subcloned into an expression construct. This construct (pcDNA3.1(+) plasmid) contained the N-terminally double-tagged (6xHis-tag directly followed by FLAG-tag) SHBs sequence of a wildtype sgtD3 reference (pcDNA3.1(+)_D3-SHBs-WT; parental HBV sequence first cloned by Nassal et al. [40] also used in Genbank accession No. MN645906) inserted into the multiple cloning site downstream of a CMV promoter and upstream of a BGH-polyA signal (both already present in pcDNA3.1(+)). The construct was used as a positive control in all experiments and as template for cloning the variant SHBs constructs. This was accomplished by exchanging nucleotides coding for amino acids 100 to 226 of SHBs. The pcDNA3.1(+)_D3-SHBs-WT backbone was amplified using primers 5′- CTT TTG TCT TTG GGT ATA CAT TTA A-3′ and 5′-GAA TCC TGA TGT GAT GTT CTC-3′, and the patient-derived sequences were amplified from the sequencing plasmids using the reverse-complementary primers 5′-GAG AAC ATC ACA TCA GGA TTC-3′ and 5′-TTA AAT GTA TAC CCA AAG ACA AAA G-3′. Recombination, transformation, and plasmid preparation were performed as described above.

### 2.3. Bioinformatics

ClustalW multiple sequence alignment was performed using BioEdit version 7.2.5.

### 2.4. Transient Transfection of HBV Expression Clones

HepG2 cells were obtained from Clontech and cultured at 37 °C and 5% CO_2_ in complete medium (DMEM; Gibco) containing penicillin/streptomycin (PAA Laboratories) and 10% fetal calf serum (FCS; Anprotec) on cell culture dishes coated with rat-tail collagen (Corning). HepG2 cells were plated on 6-well plates to reach 80–90% confluence on the day of transfection. Cells were transfected with XtremeGeneHP (Roche) following the manufacturer’s instructions. The day after transfection, cells were washed twice with PBS and cultivated in Williams’ E medium (Gibco) containing penicillin/streptomycin, 200 µM L-glutamine (Gibco), 10 mM Hepes (Gibco), and 2% FCS. On day 4 post-transfection, the cell culture supernatant was collected and cells were directly lysed in-well with an “optimized lysis buffer” described previously [41]. Supernatant and lysates were stored at −20 °C until further use.

### 2.5. Immunoblotting 

Cell lysates and crude supernatants of transiently transfected HepG2 cells were subjected to discontinuous SDS-PAGE followed by Western blotting. In brief, samples were mixed with β-mercaptoethanol (10% f.c.; Promega) and 4× Laemmli sample buffer (1× f.c.; Bio-Rad) and then boiled at 95 °C for 5 min. Samples were shortly spun down and then subjected to SDS-PAGE with a 12% separating gel. After electrophoresis, proteins were blotted onto the Immobilon-P PVDF membrane (Millipore) for 1 h at 2.5 mA/cm^2^ using a semi-dry blotting system (Biometra). Membranes were blocked using 5% skim milk powder in TBS containing 0.1% Tween-20 (TBS-T) for 1 h at RT. Subsequently, membranes were probed with primary anti-FLAG antibody (Millipore; F7425) at a 1:800 dilution in 1% skim milk powder in TBS-T overnight at 4 °C. On the following day, membranes were washed three times with TBS-T for 5 min each and then probed with secondary anti-rabbit-IgG antibody coupled to horseradish peroxidase (Cell Signaling; #7074) at a dilution of 1:2000 for 2 h at RT. Anti-α-tubulin (Sigma-Aldrich; T9026) was used as a loading control for cell lysates at a 1:1,000 dilution. Protein bands were visualized using the ClarityMax Western ECL substrate (Bio-Rad) and documented with the ChemoCam Imager ECL (Intas).

### 2.6. Deglycosylation of Proteins 

N-linked glycans of glycosylated SHBs were removed with the PNGase F Glycan Cleavage kit (ThermoFisher; A39245). Supernatants were mixed with β-mercaptoethanol (10% f.c.) and NP-40 (0.5% f.c.) and then denatured at 100 °C for 5 min. Samples were allowed to reach RT before the addition of premixed PNGase F and PNGase F buffer followed by incubation at 50 °C for 5 min and analyzed by immunoblotting as described above.

Quantification of the SHBs band was performed using the Image Studio Lite software version 5.2.

### 2.7. Diagnostic HBsAg Assays 

Based on the Western blot quantification, cell culture supernatants containing SHBs subviral particles were diluted with Williams’ E medium containing 2% FCS to reach equal amounts of SHBs before the diagnostic assays were performed. Equal amounts of SHBs were analyzed in four tests: the quantitative HBsAg (Abbott ARCHITECT HBsAg Reagent Kit 6C3643) and the corresponding HBsAg confirmatory test (Abbott ARCHITECT HBsAg Qualitative II Confirmatory Reagent Kit 2G2325) as well as the qualitative HBsAg test (DiaSorin Liaison HBsAg, code 310100) and the corresponding HBsAg confirmatory test (DiaSorin, code 310110). All tests were performed following the manufacturer’s instructions.

### 2.8. Competitive Anti-HBs Assay 

To determine the reactivity of anti-HBs with wildtype SHBs-containing subviral particles of HBV subgenotype D3, the quantitative anti-HBs assay (Abbott ARCHITECT Anti-HBs Reagent Kit, 7C1839) was used. Variant SHBs-containing cell culture supernatants were diluted with PBS to adjust variant HBsAg to equal amounts as calculated via immunoblot quantification (see above) which were equivalent to 10 IU/mL wildtype HBsAg. After the addition of 50 IU/L anti-HBs (final concentration) present in various types of human sera, samples were incubated for 1 h at 37 °C and then measured with the anti-HBs assay. The human sera came from three groups with six subjects each: (i) HBV vaccinees (anti-HBs positive, anti-HBc negative), (ii) recovered from HBV infection (anti-HBs positive, anti-HBc positive), and (iii) HBV naïve (anti-HBs negative, anti-HBc negative). 

Patient sera were obtained at the University Clinic Giessen in accordance with the local ethics committee of the Department of Medicine of the Justus Liebig University Giessen (AZ 257/18).

### 2.9. Statistical Analyses 

Unpaired *t*-test was performed using the “*t*-tests (and non-parametric tests)” tool assuming a Gaussian distribution in GraphPad Prism Version 9.2.0. Statistical significance was calculated to compare SHBs detection rates by diagnostic assays. Differences were considered significant for *p* < 0.05.

## 3. Results

### 3.1. A Patient with Detectable HBV DNA and Simultaneously High Anti-HBs

A 52-year-old male patient from Albania was referred to our outpatient clinic. He was diagnosed with chronic HBV infection at the age of 23 years. The mode of transmission was unknown. Apart from increased bilirubin (2.4 mg/dL, normal <1.2 mg/dL) due to Gilbert’s syndrome, all other liver function tests were persistently within the normal range during a one-year follow-up. A liver biopsy at the age of 52 years revealed an Ishak score of A0, B0, C2, D1, and F2 with coexistent mild steatohepatitis. Other relevant comorbidities were absent. The patient repeatedly tested positive for HBV DNA (range 64–397 IU/mL) and HBsAg with 1.68 IU/mL at the last follow-up. Anti-HBs was positive (ranging from 151 to 235 IU/L during follow-up). Total anti-HBc was positive, while IgM anti-HBc was negative. The patient showed stable HBeAg seroconversion (persistently HBeAg negative, anti-HBe positive) during the follow-up period. HDV coinfection was excluded. Based on the finding of F2 fibrosis in biopsy and the diagnosis of HBV infection with diagnostic and immune escape, antiviral treatment was initiated recently with tenofovir disoproxil.

### 3.2. Identification of Four Non-Wildtype HBV sgtD3 Variants

**Figure 1:**
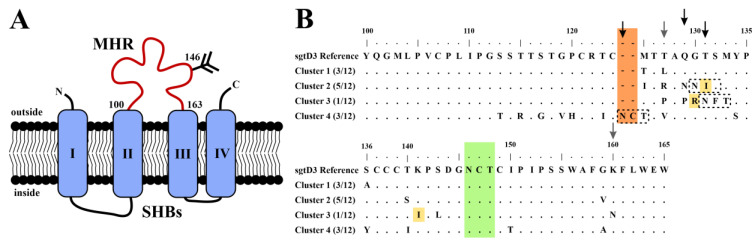
The major hydrophilic region (MHR) of mutated viral variants contains diagnostic escape mutations and additional glycosylation motifs. (**A**) The putative topology of SHBs. SHBs consists of four transmembrane domains. The MHR between transmembrane domains II and III (aa100-163) is the major target of neutralizing antibodies and is commonly associated with mutations. The N-linked glycosylation site at N146 is highly conserved. (**B**) MHR of serum-derived SHBs variants. Cluster 1 represents a subgenotype (sgt) D3 isolate (similar to the sgtD3 reference, serotype ***ayw3***) but with an unusual ***ayw4*** serotype, caused by mutation T127L. In the remaining clusters, several mutations were found: three previously described diagnostic escape mutations (cluster 2: T131I; cluster 3: G130R/K141I; yellow), a six-nucleotide insertion (cluster 4; orange), and substitutions introducing N-glycosylation motifs (clusters 2–4; boxed). The conserved N-glycosylation site (N146) is shown in green. Black arrows denote additional N-glycosylation sites. Grey arrows denote HBsAg subtype ***w***/***r*** determinant defining amino acids.

To investigate mutations affecting antigenicity of the S domain, the genome region encoding the external antigenic loop of HBsAg also termed the major hydrophilic region (MHR; Figure 1A) was amplified via PCR from serum and cloned into pcDNA3.1(+). After sequencing 12 clones, none had wildtype sequences but three were identical and most closely related to wildtype HBV of subgenotype (sgt) D3 and were termed cluster 1. Cluster 1 had three mutations in the HBsAg determinant ***a*** at M125T, T127L, and S136A. The exchange T127L results in the HBsAg subtype ***ayw4*** (or ***a4y*** according to the most recent HBsAg subtype nomenclature [42]), whereas the wildtype of sgtD3 has ***ayw3***. The majority of clones (9/12) presented other mutations in the antigenic determinants (Figure 1B; Figure S1A): The five clones of cluster 2 had the mutation T127R, and the three clones of cluster 4 had T127V, both of which are not compatible with the formation of the subtype determinants ***w1-4*** (or ***a1-4).*** The single clone of cluster 3 had a T127P exchange, which would result in ***w2*** (or ***a2yw***) which is very frequent in other HBV genotype (gt) D isolates. However, subtype determinant ***w*** requires a K160, which was mutated here to K160N. Thus, all four clusters had an inactivated or atypical ***w*** subtype determinant and the HBsAg subtype ***ay***. 

A G130N mutation of cluster 2 introduced a second potential N-glycosylation site in combination with T131I. Cluster 3 contained a T131N/M133T double mutation that leads to another additional N-glycosylation at position 131 co-occurring with the known escape mutations G130R and K141I [43]. Interestingly, the three remaining clones contained a six-nucleotide insertion between amino acids C124 and T125 (cluster 4) leading to the sequence -C^124^NCT^125^-, a previously undescribed potential N-glycosylation acceptor site. Since the surface and polymerase open reading frames overlap in the HBV genome, mutations within the MHR can similarly affect the reverse transcriptase. All relevant polymerase mutations associated with the described S variants are presented in Table 1. Importantly, none of the S mutations caused a nonsense codon in the corresponding polymerase frame, and the catalytic YMDD-motif of the RT-domain was conserved (Figure S1B).

### 3.3. All Three HBsAg Variants Bearing Additional Sequons Are Hyper-N-Glycosylated

**Figure 2:**
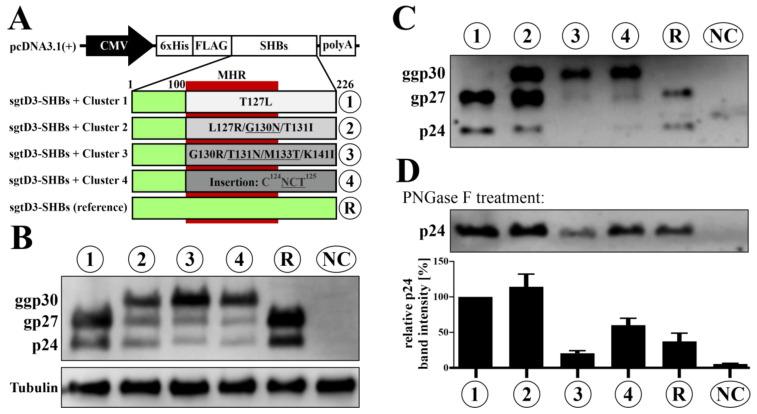
Ectopic sequons of the variant SHBs serve as N-glycosylation acceptor sites and cause hyperglycosylation of SHBs. (**A**) Construction of SHBs expression plasmids. Expression of N-terminally 6xHis- and FLAG-tagged SHBs is controlled by the CMV immediate/early promoter present in pcDNA3.1(+). The sequence encoding amino acids 100–226 (encompassing the MHR (aa100-163)) of the reference subgenotype (sgt) D3 strain was exchanged with the corresponding sequences of the viral variants. Mutations causing additional glycosylation are underlined. Representative Western blot of (**B**) intracellular and (**C**) extracellular SHBs expression pattern. HepG2 cells were transiently transfected with 6xHis-FLAG-SHBs-expression plasmids. Four days post-transfection, supernatants were collected and cells were lysed. Cell lysates/supernatants were subjected to SDS-PAGE and blotted. Membranes were probed with an anti-FLAG antibody to visualize SHBs. (**D**) SHBs in the supernatants was treated with PNGase F and subjected to Western blot as described above. Bands of deglycosylated SHBs of three independent experiments were quantified with Image Studio Lite. MHR = major hydrophilic region; NC = negative control (empty pcDNA3.1(+) was transfected); R = reference strain of sgtD3.

One sequence of each cluster (covering aa100-226) was subcloned into a FLAG-SHBs-expression construct containing the N-terminal amino acids of a reference wildtype sgtD3 strain (termed “R”; Figure 2A). Western blot analysis of cellular lysates of transiently transfected HepG2 cells showed an additional SHBs band for clusters 2–4 indicating additional N-glycosylation (ggp30) (Figure 2B). A protein band of similar molecular weight was also observed in SHBs-containing supernatants (Figure 2C). After PNGaseF treatment of supernatants, only a single band (p24) remained for all SHBs constructs, confirming additional N-linked glycosylation of clusters 2–4 (Figure 2D). While secreted SVPs of cluster 2 showed equal amounts of ggp30 and gp27, N-glycosylation of SHBs derived from clusters 3 and 4 seemed to be more efficient, since nearly exclusively double-glycosylated and little to no single- or unglycosylated SHBs was secreted. SHBs of clusters 1 and 2 seems to be more efficiently secreted than the reference SHBs (compare lanes 1, 2, and R of Figure 2B to those of Figure 2C). Clusters 3 and 4 showed higher levels of intracellular than extracellular p24 and gp27 (compare lanes 3 and 4 of Figure 2B to those of Figure 2C).

### 3.4. Ectopic N-Glycosylation of HBsAg Caused Loss of Detectability in Diagnostic HBsAg Assays

**Figure 3:**
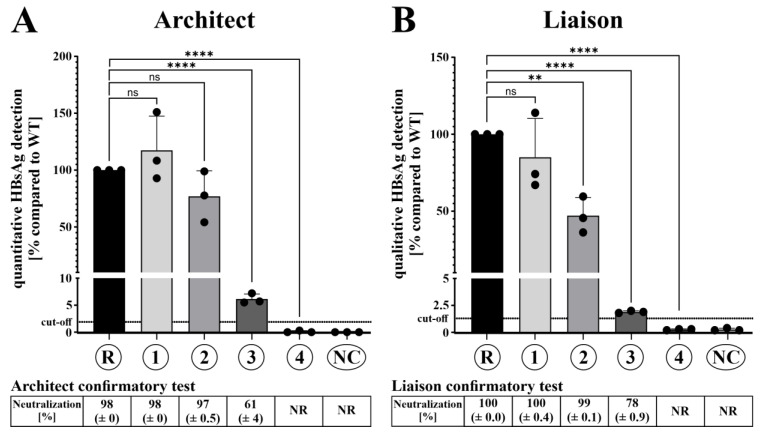
Detectability of SHBs is severely impaired by the combination of hyperglycosylation and immune escape variants. (**A**) Top panel: HepG2 cells were transiently transfected with SHBs-expressing plasmids and supernatants were collected four days after transfection. SHBs in the supernatants was adjusted to equal amounts (as quantified by immunoblot) and measured in the quantitative HBsAg assay (Abbott ARCHITECT). Bottom panel: supernatants containing equal amounts of SHBs (as quantified by immunoblot) were tested in the HBsAg confirmatory test (Abbott ARCHITECT). (**B**) Top panel: supernatants containing equal amounts of SHBs (as quantified by immunoblot) were measured in the qualitative HBsAg assay (DiaSorin Liaison). Bottom panel: supernatants containing equal amounts of SHBs (as quantified by immunoblot) were tested in the HBsAg confirmatory test (DiaSorin Liaison). All panels show the results of three independent experiments. R = SHBs of subgenotype D3 reference strain; 1/2/3/4 = SHBs containing aa1-99 of the reference strain and aa100-226 of a patient-derived sequence of cluster 1/2/3/4, respectively (also refer to Figure 2A). NC = negative control (supernatant of empty pcDNA3.1(+)-transfected cells). NR = nonreactive. ns = not significant, ** = *p* < 0.01, **** = *p* < 0.0001 (unpaired *t*-test).

Since glycosylation can affect protein antigenicity, SVPs from supernatants of transfected cells were subjected to four widely used diagnostic assays: the quantitative HBsAg and HBsAg confirmatory test (Abbott ARCHITECT) as well as the qualitative HBsAg and HBsAg confirmatory test (DiaSorin Liaison). To ensure that identical amounts of HBsAg were used in each case, the concentration of FLAG-tagged HBsAg of each sample was quantified by Western blot after complete de-N-glycosylation by PNGase F digestion (Figure 2D). Using equal amounts of untreated HBsAg from supernatants of transfected cells, the SHBs variants derived from clusters 3 and 4 showed a significant decrease in detectability compared to the wildtype sgtD3 reference (R) in both the ARCHITECT and the Liaison HBsAg assays (Figure 3). Most importantly, SHBs of cluster 4 was undetectable in both assays. Moreover, both confirmatory tests showed reduced reactivity with SHBs of cluster 3 and no reactivity for cluster 4. While SHBs of cluster 2 showed a significantly decreased detection by the qualitative Liaison HBsAg assays (Figure 3B) as well as a noticeable (but not significant) decrease in the quantitative ARCHITECT assay (Figure 3A), it was confidently recognized as true positive by both confirmatory tests.

### 3.5. Hyper-N-Glycosylated HBsAg Evades Recognition by Human Anti-HBs

**Figure 4:**
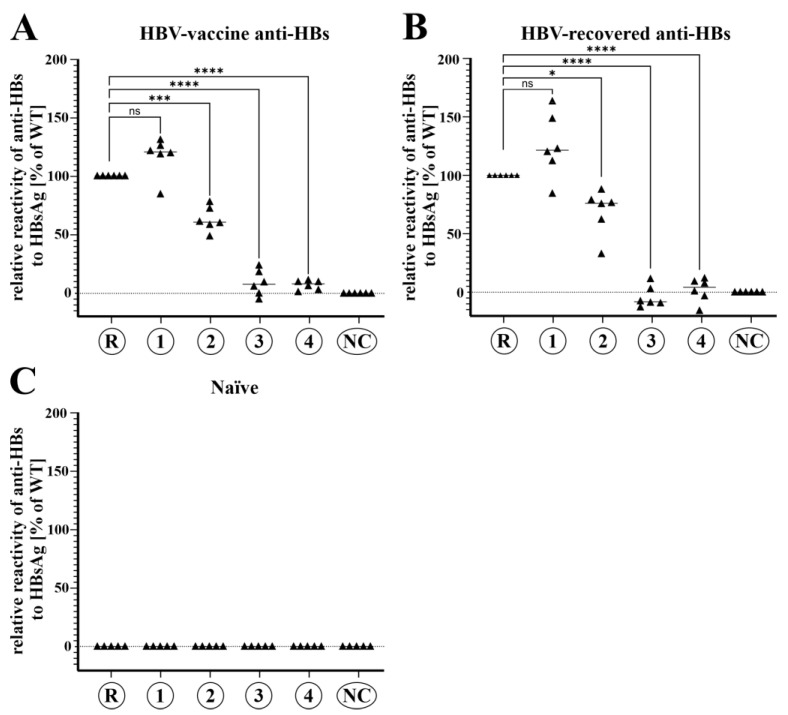
Hyperglycosylation of variant HBsAg masks epitopes recognized by anti-HBs which was induced in six human donors each by vaccination or HBV infection. SHBs within cell culture supernatants was diluted to equal amounts (corresponding to 10 IU/mL WT HBsAg) and preincubated with human anti-HBs-positive sera, containing 50 IU/L anti-HBs. After incubation, quantitative unbound anti-HBs levels were measured with the ARCHITECT anti-HBs-test (Abbott ARCHITECT). Data are shown as relative reactivity of vaccinee (**A**), convalescent (**B**), and naïve (**C**) human sera with variant HBsAg. Original data used for calculation of relative reactivities are presented in Table S1. R = SHBs of subgenotype D3 reference strain; 1/2/3/4 = SHBs containing aa1-99 of the reference strain and aa100-226 of the patient-derived sequences of cluster 1/2/3/4, respectively (also refer to Figure 2A). NC = negative control (supernatant of empty pcDNA3.1(+)-transfected cells). ns = not significant, * = *p* < 0.05, *** = *p* < 0.001, **** = *p* < 0.0001 (unpaired *t*-test).

Recognition and neutralization of HBV by anti-HBs is a key host determinant for spontaneous resolution after acute/chronic infection and prevention of infection after successful vaccination. To test immune sera for their ability to bind to variant HBsAg, we modified Abbott’s diagnostic anti-HBs-assay: The variant HBsAg was used in competition with the HBsAg incorporated in the anti-HBs-assay. Human sera were preincubated with SHBs-containing supernatant from cell culture, allowing anti-HBs to react with the cell culture-derived HBsAg before adding it to the anti-HBs assay system. Anti-HBs reacting with the variant HBsAg would then decrease the total amount of detectable anti-HBs in the samples (Table S1). The measured differences allowed the calculation of relative anti-HBs binding to variant HBsAg. Anti-HBs present in the human sera was either vaccine-induced (Figure 4A; anti-HBs positive, anti-HBc negative) or from resolved HBV-infection (Figure 4B; anti-HBs positive, anti-HBc positive), while HBV-naïve sera served as a negative control (Figure 4C; anti-HBs negative, anti-HBc negative). 

Results obtained for vaccine-induced anti-HBs binding or nonreactivity to variant HBsAg did not differ from anti-HBs originating from natural infection (Figure 4A,B). A noticeable, but not significant, elevated binding of anti-HBs antibodies to cluster 1 compared to the reference was observed (vaccine-induced: 117%/HBV-recovered: 125% relative reactivity (median values)). Recognition of cluster 2 was significantly reduced (vaccine-induced: 63%/HBV-recovered: 69%), while cluster 3 caused substantial reactivity loss for vaccine antibodies and total loss of reactivity with convalescent antibodies (vaccine-induced: 9%/HBV-recovered: −3%). Only residual reactivity was observed for both vaccine-induced antibodies and those derived after natural infection incubated with SHBs of cluster 4 (vaccine-induced: 7%/HBV-recovered: 2%). No HBs-specific antibodies could be detected with the HBV-naïve sera (Figure 4C).

## 4. Discussion

In this study, we recovered HBV DNA sequences belonging to HBV sgtD3 from the serum of an anti-HBs-positive chronic HBV carrier of Albanian descent, where HBV gtD is the most prevalent genotype [44]. The isolates retrieved formed four distinct HBV clusters of which the majority (75%) contained additional N-glycosylation motifs in the antigenic loop of HBsAg alone or in combination with known immune escape mutations and mutations changing or inactivating HBsAg subtype ***w*** subdeterminant. Importantly, cluster 4 contained a previously undescribed six-nucleotide insertion at a not-yet-described position in the MHR. We could show that all non-canonical sequons were in fact N-glycosylated in hepatic cell cultures and that hyperglycosylated SHBs isoforms were secreted efficiently. Cluster 1 without additional sequons was equally well detectable as the wildtype reference D3 HBsAg, although it contained a mutation that inactivated the subtype determinant ***w3*** common in wildtype HBV sgtD3 isolates. The detectability of secreted subviral particles of the three hyperglycosylated clusters in two diagnostic HBsAg assays (Abbott ARCHITECT and DiaSorin Liaison) was significantly decreased, with cluster 4 being completely undetectable in both. While HBsAg confirmatory tests (Abbott ARCHITECT and DiaSorin Liaison) turned out true-positive for cluster 2 and cluster 3, the results of cluster 4 were false-negative. Presented data on the reactivity of vaccine-elicited or convalescent anti-HBs antibodies revealed a significant decrease in anti-HBs binding ability for all additionally glycosylated variants. Remarkably, the binding of anti-HBs to HBsAg of clusters 3 and 4 was reduced by >90% down to only borderline detectable residual reactivity.

Glycosylation is a common immune evasion mechanism in viruses either by concealing neutralizing epitopes or through structural hindrance of the antigen processing pathway [45]. Since glycosylation is dependent on a multitude of factors besides the sole presence of an acceptor sequence [28], experimental proof of glycosylation is essential to assess clinical relevance. The ability of HBV surface proteins to allow additional N-glycosylation has been shown for both naturally occurring and purely experimental mutations [9,29]. Julithe and colleagues demonstrated that experimental sequons present at the cytosolic loop between TMD I and II (aa40-52) as well as those located close to TMD III and beyond (aa165-208) were unable to be glycosylated, while sequons located within the MHR at aa114-144 could serve as N-glycan acceptor sites with comparable glycosylation rates to those of the canonical N146-sequon [9]. 

In this study, we investigated the 133T mutant (cluster 3), one of the most frequent NLG variants [46], which is found either alone (gtA) or in combination with 131N (non-gtA; this study). We could further demonstrate that the G130N mutation (cluster 2) indeed serves as N-glycosylation acceptor site. While this mutation has been identified previously, experimental proof of its glycosylation had been lacking [29]. We also described a novel NLG sequon introduced by a six-nucleotide insertion between C124 and T125 of SHBs (cluster 4). While insertions carrying sequons are not uncommon, they are mostly found at positions 112–115 and 129–131 [26,27]. All variants tested in our study were located within the MHR, more precisely the so-called ***a*** determinant. In accordance with the mentioned studies, our data show that all additional sequons could be N-glycosylated, with double- and single-glycosylated proteins being the major component of secreted HBsAg (Figure 2C). Reduced HBsAg secretion of the reference construct, besides high intracellular expression (compared to clusters 1–4), may be attributed to isolate-specific amino acid differences outside the MHR (Figure S1).

Cluster 1 reacted as well as the reference sgtD3 HBsAg in the two diagnostic tests although it contained three mutations in the determinant ***a***, one of which at T127L replaced the ***w3*** subtype determinant with ***w4*** occurring usually in gt E and F. Interestingly, the three other clusters were also mutated at that site, though differently with R, P, or V, suggesting that the subtype determinant ***w3*** at T127 was a major target of immune selection in the studied patient. The current predominant SHBs-containing vaccines protect against all HBsAg subtypes, but asymptomatic breakthrough HBV infections in vaccinated blood donors were more often diagnosed with heterologous subtypes than with the homologous vaccine HBsAg subtype ***adw2*** [47]. It is plausible, however, that the diagnostic tests were not affected by this mutation because they were developed to detect all wildtype HBsAg subtypes equally well. The patient serum contained the mutated genomes obviously within HBV particles with most likely accordingly mutated HBsAg particles. Nevertheless, the moderately mutated virus of cluster 1 could circulate in the patient’s serum in similar amounts as the heavily mutated clusters 2–4, which might have contributed to low positive qualitative and quantitative detectability of HBsAg in diagnostic tests.

Three of the four variants tested (clusters 2, 3, and 4) showed decreased detectability in state-of-the-art HBsAg in vitro diagnostic assays, with the variant carrying the six-nucleotide insertion (cluster 4) being entirely undetectable. We presume that additional N-glycosylation is the major cause of reduced detectability in the diagnostic assays in this study, although there is one disparity: While cluster 3 (T131N/M133T) was barely detectable and cluster 4 (insertion) was false-negative, secreted subviral particles of cluster 2 (G130N) were confidently detected (Figure 3). This might be explained by the distinct secretion profiles of the SHBs glycoforms. Secreted SHBs of clusters 3 and 4 contain double-glycosylated ggp30 in the vast majority and only little to no single- and unglycosylated gp27 and p24, respectively (Figure 2C). On the other hand, subviral particles of cluster 2 contain equal amounts of ggp30 and gp27 and similar amounts of p24 when compared to the reference strain (Figure 2C, lanes 2 and R). It seems plausible to assume that unglycosylated SHBs is detected best by in vitro diagnostics, while N146-glycosylated HBsAg might also be recognized to some extent. Therefore cluster 2 subviral particles would be more readily detectable in the diagnostic assays owing to the accessibility of glycan-unshielded SHBs epitopes in contrast to the highly glycosylated HBsAg of clusters 3 and 4. The two amino acid insertion of cluster 4 most likely also affects the antigenicity of HBsAg by disrupting an essential linear epitope in aa119-125 that is targeted by several monoclonal antibodies [48,49]. In accordance with our data, a T123N glycosylation mutation at a position neighboring the cluster 4 insertion was also shown to cause a reduction in detectability by more than 99% in the ARCHITECT assay [50].

The same reasons mentioned affecting the detectability of in vitro diagnostics are also applicable to HBsAg binding with human anti-HBs. We could show that the highly glycosylated SHBs present on subviral particles of clusters 3 and 4 only shows negligible reactivity with anti-HBs from vaccinee and convalescent human sera (Figure 4). As for the in vitro assays, anti-HBs antibodies readily recognized secreted HBsAg derived from cluster 2, although with reduced capacity.

Since the NLG variant Q129N has recently been described to be horizontally transmittable despite full vaccination [26], nonreactivity of vaccine-derived anti-HBs against such HBsAg mutants could pose an increasing threat to public health. We provide further evidence that our hyperglycosylated HBsAg variants can lead to a complete loss of recognition by anti-HBs in sera from vaccinees (Figure 4) and hence might have the potential to be transmissible to HBV-vaccinated individuals, especially to those with low vaccine-response. To circumvent this problem, the use of recombinant HBV vaccines containing pre-S epitopes in addition to SHBs would be an opportunity. Especially the N-terminal region of pre-S1 is essential for infectivity and highly conserved in all HBV genotypes, thus rendering it a suitable vaccine target [51]. Promisingly, such third-generation vaccines have been shown to be superior to second-generation SHBs-only vaccines in both rate and levels of HBV seroprotection [52]. 

In regard to immune escape, we detected variants separately containing T131I (cluster 2) as well as G130R + K141I (cluster 3). G130R has been shown to abolish virion secretion while retaining anti-S antibody antigenicity [53], whereas K141E causes a severely impaired secretion phenotype [10,53,54]. This loss in viral fitness, however, is likely tolerated due to the advantage of complete antibody evasion by K141E, as this mutation has been described in vaccine failure despite protective anti-HBs levels [55]. Another study showed loss of binding of all monoclonal and polyclonal antibodies tested to mutants carrying T131I and K141E independently [56].

Interestingly, studies have shown that an HBV virion secretion deficit caused by several immune evasion mutations as well as the removal of the N146-glycan could be rescued by a concomitant M133T mutation (novel NLG site) [24,53]. Another study confirmed the beneficial effect of the N131-glycosylation and showed a similar effect for the naturally occurring 129N mutation on HBV virion secretion [26]. We found the same M133T mutation in our variant bearing G130R + K141I (cluster 3). We also observed a similar secretion impairment of this mutant as described above (Figure 2B,C): All three SHBs glycoforms (i.e., p24, gp27, and ggp30) were present intracellularly, with ggp30 being the dominant species, while gp27 and p24, which were found at similar levels, were less abundant. Secreted SVPs, on the other hand, contained mostly double- and little single-glycosylated but no unglycosylated SHBs. It is therefore likely that the combination of G130R + K141I here has the same detrimental effect on virion/HBsAg secretion as G130R + K141E. This phenotype, however, might (at least partially) be alleviated by M133T. 

We could also observe a similar secretion deficit for the mutant carrying the previously undescribed six-nucleotide insertion (cluster 4) (Figure 2B,C). The observed phenotype is in contrast to the generally described association of additional N-glycosylation with increased subviral particle secretion [9]. However, the insertion in cluster 4 also introduces an additional cysteine residue (Figure 1). Since the eight highly conserved cysteine residues present in the ***a*** determinant have an impact on intracellular trafficking/sorting and play a role as viral infectivity determinants [57], the insertion of an additional cysteine might cause incorrect folding of the antigenic loop due to changed intra- and inter-HBs disulfide bridges. A resulting misfolding of HBs and/or HBs-dimers within the ER might induce the unfolded protein response and interfere with efficient HBsAg release [58]. 

The S domain of HBsAg also plays a major role in HBV/HDV entry. It could be shown previously that additional N-glycosylations at aa129 and aa136 did not affect HDV infectivity [9]. The N-sequons described in this study are located within the same region of the MHR (aa125-136) that was found to be non-essential for infectivity [7] and are therefore unlikely to cause infectivity loss. 

Due to the compact HBV genome, mutations within the S domain can also cause simultaneous changes in the overlapping polymerase protein, specifically the reverse transcriptase domain, and thus influence viral replication. Sequence analysis of our variants revealed no premature stop codons present in the polymerase reading frame, thus likely representing replicative isolates. Due to their clinical significance, most studies have focused on the replication phenotypes of therapy resistance-associated mutations [59], and only little is known about resistance-independent mutations in the HBV polymerase. Only a few of the reverse transcriptase-associated mutations determined in this study (Table 1) have been investigated so far: We observed the K141I substitution (cluster 3) leading to rtQ149H. The rtK149R (gtC; K141E) mutation at the same position has been shown to reduce viral replication efficacy compared to WT by five log_10_ grades as measured by virion-associated HBV DNA in vitro [54]. Furthermore, the T131I mutation in our study (cluster 2) is associated with an rtN139H mutation. In a study by Jammeh and colleagues [54], the T131I mutation did not affect viral replication, which was trivial because the mutation was silent in the polymerase frame. Overall, investigation of the replication capacity of the reference strain carrying the mentioned polymerase mutations described herein is still lacking.

In our study, the patient showed sustained anti-HBs levels with detectable HBsAg. Even though anti-HBs/HBsAg co-existence has been described since the mid-1970s [60], the underlying mechanisms and effects on disease progression of this peculiar serological constellation remain elusive. The prevailing hypothesis explaining this phenomenon suggests that certain amino acid substitutions within the primary sequence of the ***a*** determinant are sufficient to alter its antigenic structure and thereby render HBsAg less recognizable by circulating (wildtype-specific) anti-HBs [61]. Consequently, circulating wildtype HBsAg would form immunocomplexes with excess of wildtype-specific anti-HBs (anti-HBs detectable), while non-wildtype HBsAg remains detectable due to evasion of humoral immune response (HBsAg detectable). This generally implies that immune pressure in any chronic patient could eventually lead to the co-existence of HBsAg/anti-HBs when quasispecies divergence increases (e.g., after anti-HBe seroconversion). This goes along with the clinical observation that the number of patients with anti-HBs/HBsAg concurrence can reach levels of up to 20–30% in large-scale cohorts [62,63].

Of note, the patient in this study showed low levels of viremia with concomitantly high levels of anti-HBs. A study by Lada et al. suggested that anti-HBs/HBsAg double-positivity favored the selection of escape mutants when viremia was low [64], while another study showed no such correlation in highly viremic HBeAg-positive patients [65]. The detected escape mutants might therefore have arisen due to the patient’s continuous low-level viremia.

The patient in this study also showed early signs of liver disease (F2 fibrosis). Several longitudinal studies have demonstrated a significant correlation between anti-HBs/HBsAg co-existence and HCC occurrence [66,67,68]. Since variants carrying additional (putative) glycosylation sites are frequent in such double-positive patients and have also been linked to HCC development [26,46], N-glycosylation mutants may play a major role in the liver disease progression of anti-HBs/HBsAg-positive chronic patients. 

Despite the chance of horizontal transmission of anti-HBs evasive variants (as mentioned above), they are not commonly spread in the general population, most likely due to the associated low viremia. However, in patients undergoing immunosuppression, variant HBV DNA levels can reach high levels, thus increasing the chances of transmission events.

We also want to acknowledge some limitations of this study. Since our experiments were performed exclusively with subgenomic constructs expressing SHBs only, the effects of the presented mutations on replication and viral protein interplay require further investigation by substitution of these sequences into a replication-competent full-length HBV plasmid. Furthermore, we did not have access to liver samples to retrieve intrahepatic HBV sequences and determine pathological changes in liver tissue such as fibrosis.

## 5. Conclusions

Overall, we described an example of the HBV quasispecies heterogeneity in terms of immune and diagnostic escape variants. We found that all variants contained an altered or deleted HBsAg subtype determinant ***w*** proving its role as a primary target of immune selection. More importantly, the majority of detected variants contained additional glycosylation sites at different positions in the MHR alone or in combination with known immune escape substitutions, highlighting the fact that several different evasion mutants can co-circulate within a patient. Lastly, the observed evasion of these hyperglycosylated HBV variants from the vaccine-induced antibodies might also pose a considerable threat to the global vaccination effort.

## Figures and Tables

**Table 1:** Mutations found in the variant sequences that are associated with diagnostic and/or immune escape in the S-ORF and their corresponding mutations in the overlapping RT/POL-ORF.

Cluster	Relevant S-ORF Mutation	Corresponding RT/POL-ORF Mutation
1	T127L *	Y135S
2	T127R **	Y135S
	G130N	R138Q
	T131I	N139H
3	G130R	R138Q
	T131N	N139K
	M133T	silent
	K141I K160N **	Q149H I169L
4	T127V ** Insertion C^124^NCT^125^	Y135C L^132^QLH^133^

* HBsAg subtype change to ***ayw4*** in comparison to the sgtD3 reference isolate of subtype ***ayw3***. ** Inactivation of HBsAg subtype determinant ***w***.

## Data Availability

The data that support the findings of this study are available on request from the corresponding author.

## Supplementary Materials

The following supporting information can be downloaded at: https://www.mdpi.com/article/10.3390/v15040838/s1, Figure S1: Alignment of amplified (partial) SHBs and polymerase sequences; Table S1: Original data and respective calculated reactivities of anti-HBs to variant HBsAg.
