# Supplementary material for: A Novel Insertion in the Hepatitis B Virus Surface Protein Leading to Hyperglycosylation Causes Diagnostic and Immune Escape
Source: Viruses. 2023 Mar 25;15(4):838. doi: 10.3390/v15040838 (PMC10144012; doi:10.3390/v15040838)
Supplement: Supplementary file 1 [file viruses-15-00838-s001.zip › viruses-2222632-supplementary.pdf]

Supplementary Information:

“A Novel Insertion in the Hepatitis B Virus Surface Protein leading to Hyperglycosylation causes Diagnostic and Immune Escape”

Felix Lehmann, Heiko Slanina, Martin Roderfeld, Elke Roeb, Jonel Trebicka, John Ziebuhr, Wolfram H. Gerlich, Christian G. Schüttler, Bernhard Schlevogt, Dieter Glebe

Dieter Glebe
Email: dieter.glebe@viro.med.uni-giessen.de

This PDF file includes:

Figure S1
Table S1

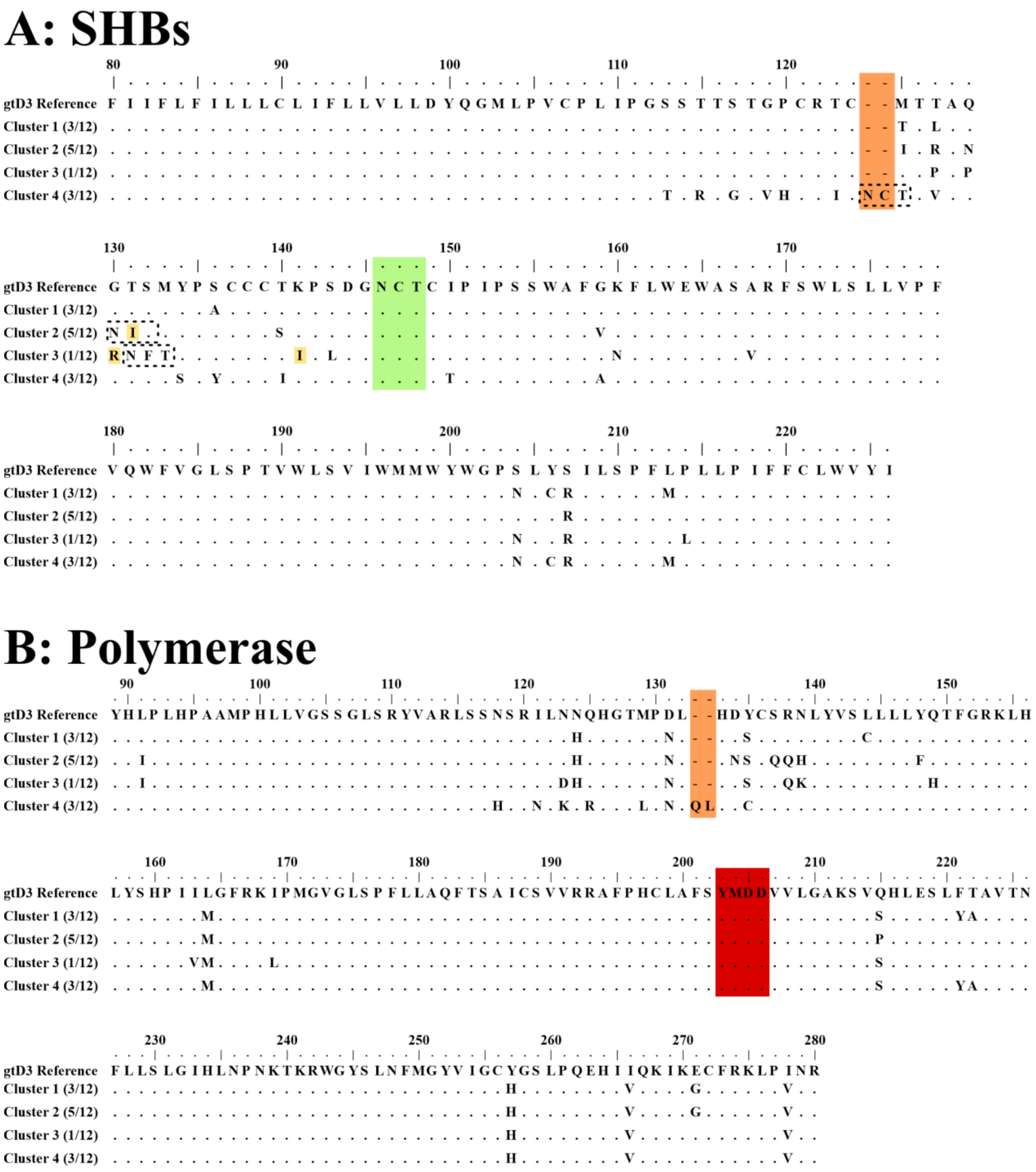

Figure S1: Alignment of amplified (partial) SHBs and polymerase sequences. (A) SHBs primary protein sequence (aa80-226). Cluster 1 represents HBV genotype D3, serotype ayw4. In the remaining clusters, several mutations were found: Three previously described diagnostic escape mutations (Cluster 2: T131I; Cluster 3: G130R/K141I; yellow), a six-nucleotide insertion (Cluster 4; orange), and substitutions introducing N-glycosylation motifs (Clusters 2-4; boxed). The conserved N-glycosylation site (N146) is shown in green. **(B)** Reverse Transcriptase domain primary protein sequence (aa89-280). The catalytic site (YMDD; red) is conserved in all isolates. The insertion of Cluster 4 introduces two amino acids (orange).

Table S1: Original data and respective calculated reactivities of anti-HBs to variant HBsAg. (A) Anti-HBs-positive human sera were diluted to reach approx. 50 IU/L anti-HBs, incubated with 10 IU/mL cell-culture-derived HBsAg for 1h, 37°C and subsequently subjected to the quantitative Architect anti-HBs-assay (Abbott). **(B)** Based on data presented in A, relative anti-HBs/HBsAg-reactivities were calculated as follows: For each serum, the individual range between negative control NC (0 %) and wildtype reference R (100 %) was used to convert measured anti-HBs values of mutant HBsAg (Cluster 1-4) into relative reactivities. Values above 100 % represent higher reactivity of HBsAg sample with anti-HBs than the reference. Accordingly, values below 0 % represent no reactivity with anti-HBs. Anti-HBs levels of all five naïve sera were 0.00 IU/L for all tested HBsAg samples and disregarded in this table.

A	Measured anti-HBs [IU/L]						
	Serum identifier	Reference (R)	Cluster 1	Cluster 2	Cluster 3	Cluster 4	NC
Vaccinees	2100001	23.00	16.15	31.83	51.91	52.92	54.97
	2144557	23.94	19.35	31.28	40.60	41.11	41.66
	2144644	26.38	23.94	31.60	39.45	38.16	39.46
	2145601	23.92	18.07	31.24	43.75	42.57	42.81
	2145578	38.84	40.89	45.65	49.70	50.62	52.14
	2146118	18.84	13.42	24.91	39.90	43.79	46.53
Recovered	2144555	25.59	22.04	31.47	42.63	41.69	41.18
	2144803	11.42	7.72	18.48	38.02	37.87	41.46
	2144893	18.09	22.49	37.08	45.53	44.19	46.34
	2145252	32.57	22.26	36.48	50.66	48.48	48.61
	2145480	31.25	21.87	33.58	52.02	48.78	50.56
	2145813	14.95	10.52	19.58	38.84	40.38	36.91

B	Relative anti-HBs/HBsAg-reactivity [% of WT]						
	Serum identifier	Reference (R)	Cluster 1	Cluster 2	Cluster 3	Cluster 4	NC
Vaccinees	2100001	100.00	121.43	72.38	9.57	6.41	0.00
	2144557	100.00	125.90	58.58	5.98	3.10	0.00
	2144644	100.00	118.65	60.09	0.08	9.94	0.00
	2145601	100.00	130.97	61.25	-4.98	1.27	0.00
	2145578	100.00	84.59	48.80	18.35	11.43	0.00
	2146118	100.00	119.57	78.08	23.94	9.90	0.00
Recovered	2144555	100.00	122.77	62.28	-9.30	-3.27	0.00
	2144803	100.00	112.32	76.50	11.45	11.95	0.00
	2144893	100.00	84.42	32.78	2.87	7.61	0.00
	2145252	100.00	164.28	75.62	-12.78	0.81	0.00
	2145480	100.00	148.58	87.93	-7.56	9.22	0.00
	2145813	100.00	120.17	78.92	-8.79	-15.80	0.00
